# Adaptation and validation of the patient assessment of chronic illness care in the French context

**DOI:** 10.1186/1472-6963-14-269

**Published:** 2014-06-19

**Authors:** Nicolas Krucien, Marc Le Vaillant, Nathalie Pelletier-Fleury

**Affiliations:** 1 HERU, University of Aberdeen, Polwarth Building, Foresterhill, Aberdeen, AB25 2ZD, UK; 2 CERMES3, CNRS UMR 8211 – INSERM U988, 7 rue Guy Moquet, 94 801 Villejuif cedex, France

## Abstract

**Background:**

Chronic diseases are major causes of disability worldwide with rising prevalence. Most patients suffering from chronic conditions do not always receive optimal care. The Chronic Care Model (CCM) has been developed to help general practitioners making quality improvements. The Patient Assessment of Chronic Illness Care (PACIC) questionnaire was increasingly used in several countries to appraise the implementation of the CCM from the patients’ perspective. The objective of this study was to adapt the PACIC questionnaire in the French context and to test the validity of this adaptation in a sample of patients with multiple chronic conditions.

**Methods:**

The PACIC was translated into French language using a forward/backward procedure. The French version was validated using a sample of 150 patients treated for obstructive sleep apnea syndrome (OSAS) and having multiple chronic co-morbidities. Several forms of validity were analysed: content; face; construct; and internal consistency. The construct validity was investigated with an exploratory factorial analysis.

**Results:**

The French-version of the PACIC consisted in 18 items, after merging two pairs of items due to redundancy. The high number of items exhibiting floor/ceiling effects and the non-normality of the ratings suggested that a 5-points rating scale was somewhat inappropriate to assess the patients’ experience of care. The construct validity of the French-PACIC was verified and resulted in a bi-dimensional structure. Overall this structure showed a high level of internal consistency. The PACIC score appeared to be significantly related to the age and self-reported health of the patients.

**Conclusions:**

A French-version of the PACIC questionnaire is now available to evaluate the patients’ experience of care and to monitor the quality improvements realised by the medical structures. This study also pointed out some methodological issues about the PACIC questionnaire, related to the format of the rating scale and to the structure of the questionnaire.

## Background

Chronic diseases are major causes of disability worldwide and prevalence is rising [1]. In France, the number of patients officially identified as having at least one chronic condition rose from 3.7 million in 1994 up to 9 million in 2008, and could reach 12 million in 2015 [2]. In addition to the medical burden, the growth of chronic conditions places a high financial burden on the healthcare systems. In 2007, the amount of public insurance reimbursements to those patients was estimated at 80 billion euros [3].

Despite these large expenditures, patients suffering from chronic conditions do not always receive optimal care [4,5]. There is evidence that a long-term, structured, and proactive approach can help to reduce the burden of chronic diseases, but the organization of medical care is still mainly oriented toward acute conditions [6]. The management of chronic conditions is often more complex than that of acute conditions, because i) it involves several actors practicing in different sites, ii) the patient plays a key role in the treatment effectiveness through his/her health habits and regular compliance, and iii) given the long-term nature of the chronic conditions, chronic care has to deal with symptoms and to anticipate further complications. Then acute care organization is to some extent inappropriate to adequately manage patients with chronic conditions and to achieve effective and high quality healthcare system [7,8].

In practice, the management of chronic conditions is mainly based on primary care and especially on services dispensed by the general practitioner (GP) [9]. In most countries, including France, the GP usually acts as a gatekeeper and he/she is the first health professional to adapt to the growing burden of chronic conditions [10]. The chronic care model (CCM) has been developed to help GPs make the transition from acute care to chronic care [11] and it is widely adopted in several countries as a framework to reform the organization of GP care. The CCM describes a set of 6 elements designed to optimize the management of chronic illness, namely community linkages, organizational support, self-management support, delivery system design, decision support, and clinical information systems. The goal of treatment aligned with CCM principles is to create an informed, activated patient interacting with a prepared, proactive practice team, resulting in productive encounters and improved outcomes [12]. There is evidence that interventions containing at least one CCM element could lead to improved patient care and health outcomes [13-15]. For example, patients suffering from chronic obstructive pulmonary disease and benefiting from a care including CCM components showed lower rates of hospitalizations, and shorter hospital stays [16].

In line with the increasing use of the CCM to improve the quality of the chronic care and the recent trend to make healthcare systems more accountable for the patients’ needs, Glasgow et al. have developed the "Patient Assessment of Chronic Illness Care" (PACIC) questionnaire to appraise the implementation of the CCM from the patients’ perspective [17]. To date the PACIC questionnaire has been used in several countries to evaluate the delivery of CCM activities for a variety of chronic conditions (Table 1), but there is no version available in French language. In addition, validation studies have shown mixed evidence regarding the data quality and the properties of the PACIC scales [18,19].

**Table 1:** Literature review of the studies using PACIC questionnaire (N = 22)

**Study**	**Country**	**Sample size**	Chronic condition(s)*	**Factor analysis**	**Gender (% male)**	**Age (mean + SD or %)**	**PACIC mean score (SD)**
Maindal et al. [37]	Denmark	481	DM	Confirmatory	60%	66,4 (10,7)	2,83 (-)
Rosemann et al. [25]	Germany	236	OA	Exploratory	45%	-	2,44 (1,1)
Schmittdiel et al. [46]	USA	4,108	RESP; DM; HF; CAD; Pain	No	50%	61,9 (14,7)	2,7 (1,1)
Vrijhoef et al. [44]	Netherlands	89	COPD; HF; Arthritis; Geriatric disorders	No	49%	56% " > 65 years"	2,6 (-)
Taggart et al. [42]	Australia	2,438	DM; IHD	Exploratory	47%	63,2 (-)	3,01 (1,07)
Taggart et al. [42]	Australia	914	DM; IHD	Exploratory	43%	57,4% " ≥ 60 years"	3,07 (1,06)
Aragones et al. [27]	USA (Spanish language)	100	DM	Confirmatory	21%	-	3,17 (0,82)
Wensing et al. [41]	Netherlands	165	DM; COPD	Exploratory	53%	68 (10,3)	2,9 (1)
Rick et al. [39]	UK	2,439	Various	Confirmatory	51%	51,7% " ≥ 65 years"	2,4 (0,87)
Ludt et al. [47]	Europe	1,745	CVD	No	68%	67,8 (9,9)	2,84 (-)
Szecsenyi et al. [45]	Germany	1,399	DM	No	46%	70,3 (8,5)	2,86 to 3,21 Glasgow
Glasgow et al. [24]	USA	266	Various	Confirmatory	44%	64,2 (10,5)	2,6 (1)
Mackey et al. [26]	USA	1,823	DM	No	35%	51,5 (16,7)	3,05 (1,19)
Glasgow et al. [17]	USA	363	DM	No	53%	64,1 (11,9)	3,2 (0,9)
Cramm & Nieboer [38]	Netherlands	1,321	CVD	Confirmatory	53%	63,8 (10,2)	2,68 (0,86)
Cramm & Nieboer [38]	Netherlands	917	COPD	No	52%	63,99 (10,19)	2,89 (0,89)
Cramm & Nieboer [48]	Netherlands	1,570	CVD	No	53%	66,11 (10,57)	2,77 (0,86)
Cramm & Nieboer [50]	Netherlands	892	COPD	No	54%	66.1 (10.6)	2.89 (0.9)
Cramm & Nieboer [49]	Netherlands	283	Older people (>65 years)	Confirmatory	46%	75.8 (6.8)	-
Cramm & Nieboer [51]	Netherlands	548	COPD	No	54%	69.1 (10.2)	2.85 (0.92)
Gugiu et al. [19,28]	USA	529	DM	Confirmatory/ Exploratory	52.70%	63,4 (-)	-
Spicer et al. [18]	New Zealand	307	DM; CDV; RESP; Pain	-	56%	68,4 (-)	-
McIntosh [40]	Canada	936	-	Confirmatory	-	_	-

*DM: Diabetes; **CVD**: Cardiovascular disease; **OA**: Osteoarthritis; **CAD**: Coronary artery disease; **COPD**: Chronic obstructive pulmonary disease; **IHD**: Ischaemic heart disease; **HF**: Heart failure; **RESP**: Respiratory problems (e.g. Asthma).

The objective of this study was to translate the PACIC questionnaire into the French language and to test the validity of this adaptation in a sample of patients suffering from obstructive sleep apnea syndrome (OSAS) and other frequent chronic conditions. We were especially interested in the validation of the PACIC survey in a sample of patients suffering from multiple chronic conditions, because in practice GPs face a growing number of these patients and most of the time these patients require more complex medical care.

The remainder of this study is divided into four sections. Section 1 presents the methods used to translate and validate the PACIC questionnaire in the French language. Section 2 presents the results of the French-PACIC questionnaire and the level of achievement of the CCM recommendations in the French context. Section 3 discusses the results of this study. Finally, section 4 provides concluding remarks on this adaptation and validation of the PACIC questionnaire in French context.

## Methods

### Sample of patients with multiple chronic conditions

#### Definition of the sample size

The sample size was defined according to Hatcher (1994) who recommended that the number of subjects should be 5 times the number of variables (equivalently a 5:1 subject-to-variable (STV) ratio), with a minimum of 100 subjects [20]. In a recent 2-years literature review of the published factorial analyses (exploratory factor and principal components analyses), Costello & Osborne [21] showed that 40.5% of the studies used a sample size with a STV ratio lower than 5:1 and 36.8% of the studies with a ratio larger than 10:1. In a simulation study, MacCallum et al. (1999) showed a perfect recovery of population factor structure with a 3:1 ratio (i.e. 60 subjects and 20 variables) [22]. In this study, we expected a maximum number of 20 items. Then a minimal sample size of 100 respondents was required. Finally we decided to recruit 150 individuals to obtain a large enough sample after accounting for missing values.

#### Identification of the patients suffering from multiple chronic conditions

The OSAS chronic condition was used as a way to identify patients suffering from multiple chronic conditions already analysed in the international literature (e.g. Diabetes). As shown in this study, on average the recruited patients suffered from 2.2 chronic conditions in addition to their OSAS. In addition, at least in the French context, the GP is usually not involved in the management and treatment of the OSAS, which is largely treated by hospital-based pulmonary specialist. Then the OSAS condition was assumed to be of minor influence (if any) on the patients’ perception of the quality of the chronic care delivered by their GP.

To rule out a potential influence of the OSAS condition on the results and to be included in the validation study, patients had to meet three eligibility criteria. First, they had to suffer from at least 1 chronic condition in addition to their OSAS to be considered as patients with multiple chronic conditions. Second, they had to have experienced at least 1 GP visit in the last 6 months. This criterion was used to ensure that respondents had a minimal experience of the GP care. Third, they had to comply with their OSAS treatment for at least 1 year. Under these conditions the OSAS become asymptomatic and the treatment is well accepted by the patients. This last criterion was used to ensure a homogeneous group of patients in terms of OSAS treatment.

#### Recruitment of the patients and administration of the survey

The respondents were consecutively recruited between October 2010 and June 2011 in a hospital setting to avoid biases related to recruitment in GP office when the purpose of the study was to ask patients about their GP care (e.g. responses heavily based on the last consultation experienced, respondents’ inhibition) [23]. The patients were explicitly told that the survey was about the medical services delivered by their GP to manage their chronic conditions. This perspective was facilitated because the patients were first asked about their GP consultations and their chronic conditions to ensure their eligibility. Then when they answered the PACIC questions they were explicitly told to refer to the above mentioned chronic conditions and GP consultations. The data collection was done using a self-completed survey that was administered by a trained nurse during a follow-up visit for OSAS. In addition to the PACIC items, the survey included questions about respondents’ individual characteristics. The patients completed the survey in the waiting room. The content and design of this study were checked by a local ethic committee (*Comité de Protection des Personnes Ile-de-France VI, Pitié Salpétrière, France*), which provided agreement for the conduct of the study. Written informed consent was obtained from the patients for the publication of this report and any accompanying images.

### Initial version of the PACIC questionnaire

The initial version of the PACIC questionnaire was developed by Glasgow et al. in the United-States with 283 patients suffering from various chronic illnesses [17]. This initial version consisted in 20 items divided into 5 pre-specified dimensions of the GP care as recommended by the CCM (i.e. patient activation; delivery system/practice design; goal setting/tailoring; problem solving/contextual; follow-up/coordination). The 5 PACIC subscales did not match perfectly with the 6 elements of the CCM because some aspects of the model, such as clinical information systems and organizational support, are generally not visible to patients. These 20 items were chosen from 46 items designed by national experts on chronic illness care and the CCM.

The achievement level of each of these items was rated by patients on a 5-point rating scale with the following anchors: Almost never (1), Generally not (2), Sometimes (3), Most of the time (4), Almost always (5). However, in the literature, studies have imposed marginal changes on the format of the initial rating scale. Glasgow et al. [24] and Rosemann et al. [25] stated that "*Each item was scored on a 5-point scale ranging from 1 (no or never) to 5 (yes or always)*". Mackey et al. [26] used a 5-points scale ranging from "*None of the time*" through "*Always*". Aragones et al. [27] used a 5-points scale with the following anchors, which are also those of the current official version of the PACIC survey in English language: *None of the Time, A Little of the Time, Some of the Time, Most of the Time and Always*. Other studies have imposed changes on the rating scale. Gugiu et al. [19,28] employed an 11-point percentage scale ranging from 0% to 100% by units of 10% with two end points labelled "Never" and "Always". In this study, the 5-points rating scale was based on the following anchors: Never (1), Rarely (2), Sometimes (3), Most of the time (4) and Always (5).

### Translation and adaptation of the questionnaire

#### Translation

According to recommendations from the international literature [29], the translation of the PACIC items into the French language was performed following a forward/backward multi-steps procedure. First, each author translated the list of items into the French language without discussing this with the other authors. Second, the lists of translated items were compared and disagreements were discussed, if necessary a 4^th^ expert was solicited to reach a consensus. Third, the agreed list of translated items was presented to an external team of health professionals and medical researchers, mainly to assess the contextual relevancy of the items and the understandability of their translation. Fourth, external researchers were solicited to perform a backward translation (from French to English language) of the items. This multi-steps process appears to be in line with the World Health Organization (WHO) recommendations to translate and adapt an English instrument in different languages [WHO, 2008]. This approach putted emphasis on conceptual rather than literal (i.e. word-to-word) translations. For this purpose, experts involved in the translation process were required to have good knowledge of the French and English languages, to be familiar with the field of research and to be able to translate the concepts avoiding the use of any technical jargon.

#### Adaptation

The French version of the PACIC questionnaire was piloted with 10 patients suffering from chronic illnesses by using individual interviews. The patients were interviewed and were asked to explain their responses (i.e. think aloud approach). This pilot study was performed to ensure the adequacy of the questionnaire content to the French context. At this stage, patients had opportunities to suggest new items, to modify the translated items, and to suggest deletion of some of them.

### Validation of the questionnaire

The validity of the French PACIC questionnaire was tested following three steps: face validity; construct validity; and internal consistency.

#### Face validity

The notion of face validity refers to the extent to which a test is subjectively viewed as covering the concept it is supposed to measure [30]. In practice, the face validity of the PACIC is usually assumed to be verified because the PACIC questionnaire is directly derived from the CCM components. In this study, this assumption was tested by asking a pilot sample of patients with chronic illnesses about the relevancy of the PACIC items to describe their GP care experience.

In addition, the face validity was investigated through analysis of the data quality using descriptive statistics and correlation measures. The descriptive statistic included mean, standard deviation (SD), percentage of missing values, extent of ceiling and floor effects, and normality measures (i.e. Kurtosis, Skewness, and Anderson-Darling statistics). The floor and ceiling effects referred to the percentage of respondents using the most extreme (upper or lower) response categories. In practice, a percentage larger than 20% was associated with floor/ceiling effects [20].

#### Construct validity

The construct validity was tested using exploratory factor analysis (EFA). Given the ordinal and non-normal nature of the data collected using a 5-points rating scale, conventional methods of EFA which rely on the Pearson correlations and/or maximum likelihood techniques were deemed inappropriate. Actually, Pearson correlation coefficients are likely to underestimate the true correlations between the PACIC items and maximum likelihood approach is based on the unwarranted assumption of data normality. In this study, the factor structure of the CCM was explored using the Mplus software allowing both to handle ordered categorical variables and to use appropriate factoring method (i.e. robust weighted least squares [WLSMV]) [31].

The Kaiser’s measure of sampling adequacy (K-MSA) was used to evaluate suitability of data for factor analysis [32]. A value between 0.5 and 1 suggested that the sample was appropriated for a factor analysis. Second, the factors were selected following a parallel analysis implemented in SAS using a modified version of the Kabacoff’s code [33]. This approach is based on the idea that non-trivial factors should have eigenvalues greater than those derived from randomly generated data with the same number of variables and sample size. In this study, 1,000 datasets were simulated. Each dataset had the same dimension than the actual dataset (i.e. 18 variables/columns and 148 respondents/rows). For each simulated dataset, the variables were assumed to be normally distributed with a mean = 0 and variance = 1. Each time, the complete factorial solution is computed (i.e. the number of retained eigenvalues is equal to the maximum number of variables in the analysis). Then we obtained 1,000 sets of 18 eigenvalues computed on simulated datasets. Finally, the 95th percentile of simulated eigenvalues was computed and used as a lower bound to identify the number of actual eigenvalues to select. Only the actual eigenvalues higher than the 95th percentile of the simulated eigenvalues were considered for the final analysis. Despite being considered as the reference approach to identify the number of factors to retain, the applicability of the parallel analysis is somewhat limited when the data are non-normally distributed. In this study, the results from the parallel analysis assuming normally distributed data were taken as an acceptable approximation of those that would be obtained with a parallel analysis relaxing the Normality assumption.

A Geomin rotation procedure was used to simplify the interpretation of the factors. The Geomin rotation is an oblique rotation method allowing the factors to be correlated. The Geomin procedure produces two types of matrix: the ‘rotated loadings matrix’ which includes linear combination of variables that make up the factor, and the ‘factor structure matrix’ which includes the correlation coefficients between the variables and the factors, thus indicating which items measure the factors best. In this study, given the limited number of factors (i.e. 2), the factors were interpreted according to the factor structure matrix only.

The quality of the factorial solution was evaluated with the root mean squared error of approximation (RMSEA) was used. It measures the average amount of misfit between the model and the data. This measure is obtained by investigating the factorial solution in a confirmatory factor analysis which analyses the likelihood of observed data given the assumed model/structure. In practice, it is usually admitted that a value of the RMSEA lower than 0.05 indicates a ‘good’ fit, [0.05-0.08] a ‘reasonable’ fit, [0.08-0.1] a ‘mediocre’ fit and larger than 0.1 a ‘poor’ fit [34].

#### Reliability

The internal consistency of the French version of the PACIC was assessed using both Cronbach alpha and ordinal alpha coefficients. Theoretically, the Cronbach alpha is only appropriate when variables are continuous, and it has been shown that Cronbach-α is negatively biased when it is used to measure the reliability of ordinal variables [21]. However this measure is frequently used in practice and leads to valid results despite data that are highly skewed. According to the literature a value > 0.7 might be considered as acceptable [24].

More recently, the ordinal alpha coefficient has been suggested as a better measure when the assumption of normality is violated [35]. In their study based on simulated data, Zumbo et al. [35] concluded that "*ordinal alpha provides consistently suitable estimates of the theoretical reliability, regardless of the magnitude of the theoretical reliability, the number of scale points, and the skewness of the scale point distributions. In contrast, Cronbach alpha is in general a negatively biased estimate of reliability for ordinal data*".

### Implementation of the CCM in the French context

Once the French-version was validated, the responses were used to analyze the implementation of the CCM recommendations in GP care as perceived by the patients. Currently, there is no indication of the best way to treat missing values to compute PACIC score(s). We used a conservative approach excluding from the analysis the respondents who did not fulfill at least 75% of the items. According to Glasgow et al., it was more reliable to focus on the overall PACIC score (i.e. over all the items) rather than on the scores by dimension [17]. Consequently, we constructed a composite score using a confirmatory factor analysis on the 18 items responses, and performed an analysis of variance (ANOVA) of this overall PACIC score using the following patients’ characteristics as covariates: gender, age, self-reported health, number of chronic conditions, and number of GP consultations per year. The data management and statistical analyses were performed using SAS 9.2 and MPlus software.

## Results

### Sample of patients with multiple chronic conditions

On average the 150 recruited patients had 2.23 chronic conditions (SD:1.04) in addition to their OSAS. The 3 most frequent chronic conditions were hypertension (27.7%), diabetes (17.5%), and cardiovascular diseases (12.5%). Regarding their medical care experience, on average the patients had consulted a GP 4.15 times during the last 12 months (SD:2.07; Median = 4) and a specialist 3.11 times (SD:2.36) for a total of 7.26 medical consultations per year (SD:3.37; Median = 6). Other individual characteristics are summarized in Table 2.

**Table 2:** Characteristics of the respondents (N = 150)

**Characteristic**	**%**
**Number of chronic conditions**	
2	25.3
3	40.6
4+	34.1
**Gender**	
Male	71.3
Female	28.7
**Age**	
< 60 years	29.5
[60–65] years	32.2
[66–70] years	18.1
> 70 years	20.2
**Self-reported health**	
Excellent	2
Very good	9.3
Good	36
Satisfactory	44
Bad	8.7
**Same GP since**	
< 1 year	5.4
[1-5] years	16.6
> 5 years	78
**Last GP consultation**	
< 15 days	20.1
[15-30] days	24.1
[1-3] months	37.5
> 3 months	18.3
**Medical structure of the GP**	
GP working alone in the structure	52.7
GP working with other GPs in the same structure	40.7
GP working with other GPs and health professionnals in the same structure	5.3

### Translation and adaptation of the questionnaire

#### Translation

The forward/backward translation procedure was successfully applied. The comparison of the backward translated version to the official PACIC survey in English language showed two systematic differences. Instead of using an indefinite pronoun (e.g. "one told me") and the past tense (e.g. "I was informed") to formulate the questions/items, the translated version was based on a GP-oriented approach (e.g. "the GP told me") and present tense (e.g. "I am informed"). These changes arose because during the process of translation and piloting the indefinite pronoun approach was deemed too vague to assess the quality of the care delivered by the medical team. In addition, the translated version focused on the GP role because in the French context this health professional is the main provider of primary care and most of the patients have never experienced something else. The comparison of the forward translation in French language with another untested French version of the PACIC survey revealed no major discrepancy between the items [36]. These analyses provide support for the validity of our multi-step translation process.

#### Adaptation

The pilot study led to two major changes in the translated version of the PACIC. First, the set of 20 items was reduced to one of 18 items because 2 items were perceived as redundant by the patients. The items "Given me a written list of things I should do to improve my health" and "Help me to set specific goals to improve my eating or exercise" were merged into one item "Help me to list things to do to improve my health". In the same way, the items "Contact me after a visit to see how things were going" and "Ask me how my visits with other doctors were going" were also merged into only one item "Contact me to know how things were going". Actually the respondents were able to detect the differences between these closely related items, but according to their medical experience, they explained that in practice they had no way to distinguish one item from each other. Alternatively, these apparently redundant items would have been kept in the PACIC survey, but they would have been perfectly correlated and this would imply methodological difficulties in the factorial analysis.

The respondents had the opportunity to suggest new items and this did not led to identification of new items, thus suggesting that the initial pool of 20 items was comprehensive enough to describe the patients’ experience of GP care.

### Validation of the questionnaire

#### Face validity

The French-PACIC items seemed relevant to describe respondents’ GP care experience. Respondents did not face any difficulties completing the questionnaire.

Regarding the extent of missing values, only 4 respondents out of 150 fulfilled less than 75% of the items and were then excluded from the analysis. It remained 146 respondents who provided 2,324 observations out of 2,336 possible, a missing value rate of 0.5%. This very high rate of completion might be partly attributable to the administration process of the survey and to the setting in which the survey took place. This 75% cut-off value was initially selected according to minimal requirements for the factorial analysis. Without running a formal sensitivity analysis, our results are expected to be robust to changes in this cut-off value because 91.3% of the respondents completely filled the survey (i.e. 0 missing value) and 96% completed it with less than 10% of missing values. Then, given the sample size of this study, including/excluding the respondents with a rate of missing values larger than 10% is not expected to change the results.

Looking at the % of ‘Never’ and ‘Always’ ratings (Table 3), 13 items exhibited potential floor effect (i.e. % "Never" > 20%), with 8 items having more than 50% of their observations concentrated on this modality. Only 4 items are susceptible to ceiling effect with more than 20% of their observations pertaining to the "Always" modality. None of the items suffer from both floor and ceiling effects. These results suggested non-normal distributions and this was confirmed by the Kurtosis and Skewness measures that depicted in most of cases positively skewed (i.e. S > 0) and leptokurtic (i.e. K < 0) distributions.

**Table 3:** Descriptive analysis of the responses to the PACIC questionnaire

**Item**	**% missing values**	**% never**	**% always**	**Median**	**Kurtosis**	**Skweness**
1. The GP asks about my ideas when we made a treatment plan	2.0%	39.5%	14.3%	3	-1.49	0.22
2. The GP helps me to make a treatment plan that I could do in my daily life	4.0%	34.7%	13.6%	3	-1.44	0.19
3. The GP gives me a copy of my treatment plan	2.0%	83.7%	2.7%	1	6.54	2.73
4. The GP gives me choices about treatment to think about	2.7%	52.4%	8.2%	1	-0.29	1.00
5. The GP thinks about my values and traditions when s/he recommends treatments to me	4.0%	73.5%	5.4%	1	1.94	1.81
6. The GP helps to plan ahead so I could take care of my illness even in hard times	2.0%	27.2%	11.6%	3	-1.27	0.14
7. The GP told me how my visits with other types of doctors help my treatment	3.3%	14.3%	51.7%	5	-0.54	-1.00
8. The GP shows me how what I do to take care of my illness influence my condition	3.3%	12.2%	42.9%	4	-0.54	-0.87
9. The GP asks me to talk about my goals in caring for my illness	3.3%	50.3%	6.1%	1	-0.05	1.07
10. The GP asks me questions about my health habits	2.7%	19.0%	18.4%	3	-1.06	-0.32
11. The GP helps me to list things that I can do to improve my health	2.0%	59.2%	8.2%	1	-0.35	1.05
12. The GP refers me to a dietician, health educator, or counsellor	2.7%	57.8%	4.1%	1	0.88	1.35
13. The GP encourages me to go to a specific group to help me with my chronic illnesses	2.0%	86.4%	0.7%	1	9.00	3.00
14. The GP encourages me to attend programs in the community that could help me	2.7%	92.5%	0.0%	1	14.60	3.88
15. The GP asks me how my chronic illnesses affect my life	2.0%	32.7%	12.2%	2	-0.89	0.60
16. The GP asks me to talk about any problems with my medicines or their effects	3.3%	15.6%	36.1%	4	-1.12	-0.49
17. The GP asks me if I am satisfied with the organisation of my care	2.0%	44.9%	14.3%	2	-1.33	0.46
18. The GP contacts me to know how things are going	2.0%	16.3%	33.3%	4	-0.99	-0.64

GP: General practitioner.

#### Construct validity

**Figure 1:**
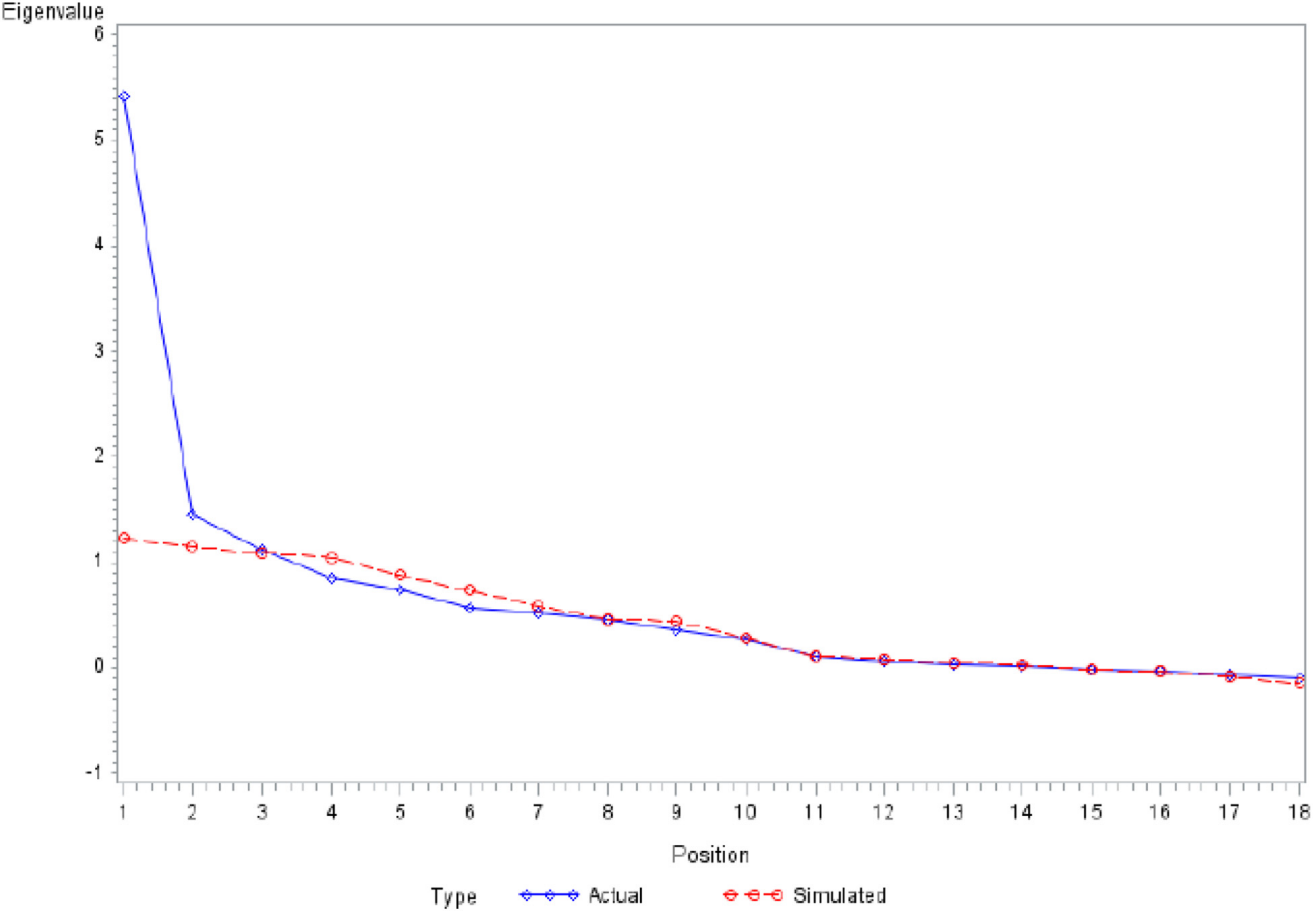
Results of the parallel analysis to identify the number of factors.

First, looking at the K-MSA values (Table 4) only 2 items showed a bad level of adequacy for a factor analysis, namely "The GP encourages me to go to a specific group to help me with my chronic illnesses" and "The GP encourages me to attend programs in the community that could help me". The overall measure is about 0.82 and thus suggested that the observations were suitable for the factor analysis.Second, the parallel analysis led to the identification of a factorial solution in 2 factors (Figure 1). It should be noted that these 2-factors solutions also satisfied the standard Kaiser criterion for the factors identification (i.e. eigenvalues > 1).

**Table 4:** Measure of the sample adequacy (MSA) for factor analysis

**Item**	**Kaiser MSA**	**Group**
1	0.84	*Very good*
2	0.85	*Very good*
3	0.84	*Very good*
4	0.85	*Very good*
5	0.73	*Good*
6	0.85	*Very good*
7	0.85	*Very good*
8	0.82	*Very good*
9	0.83	*Very good*
10	0.84	*Very good*
11	0.78	*Good*
12	0.8	*Very good*
13	0.67	*Bad*
14	0.65	*Bad*
15	0.9	*Excellent*
16	0.91	*Excellent*
17	0.82	*Very good*
18	0.78	*Good*
**Overall**	**0.82**	***Very good***

Kaiser MSA: ≥ 0.9: Excellent; [0.8-0.9]: Very good; [0.7-0.8]: Good; [0.6-0.7]: Bad; [0.5-0.6]: Very bad; < 0.5: Inacceptable.

Third, the Geomin rotation procedure led to a substantial inter-factors correlation of 0.415, thus suggesting that the two dimensions of the PACIC cannot be seen as independent dimension. Looking at the factor structure matrix (Table 5), each item was assigned to only one factor with the highest contribution (i.e. magnitude of the coefficients). According to this approach, the 1^st^ factor would be best described by 10 items and the 2^nd^ factor by 8 items. However, given the somewhat high level of inter-correlation between the two factors, some items {3;4;9;10;11;15} seemed to be well accounted for by both factors. The first factor referred mainly to items describing efforts made by the GP to customise the chronic care according to the patients’ needs and values (e.g. asking about ideas when making the treatment plan; making a treatment plan suitable in patients’ daily life; taking into account patients’ values when recommending a treatment; taking into account the patients’ health habits; making a treatment plan allowing for changes in patients situation) and to items describing the interest of the GP in the medical experience of the patients (e.g. asking how the chronic conditions affect the patients’ life; asking for problems with the medicines; asking if patients are satisfied of the chronic care; contacting patients to know if things are doing well). The second factor referred to the actions taken by the GP to implement a collaborative chronic care with the patients (e.g. giving a copy of the treatment plan; giving choice about treatment; providing a list of things to do) and other professionals (e.g. referring to a dietician; encouraging to go to support group; encouraging to use community/social programs).

**Table 5:** Results of the factor analysis

	**Item**	**Factor 1**	**Factor 2**	**Dimension**
**Factor 1**	2. The GP helps me to make a treatment plan that I could do in my daily life	0.707	0.416	Customisation
	5. The GP thinks about my values and traditions when s/he recommends treatments to me	0.498	0.373	Customisation
	6. The GP helps to plan ahead so I could take care of my illness even in hard times	0.730	0.301	Customisation
	7. The GP told me how my visits with other types of doctors help my treatment	0.544	0.048	Experience
	8. The GP shows me how what I do to take care of my illness influence my condition	0.717	0.208	Cooperation
	10. The GP asks me questions about my health habits	0.649	0.505	Customisation
	15. The GP asks me how my chronic illnesses affect my life	0.710	0.502	Experience
	16. The GP asks me to talk about any problems with my medicines or their effects	0.589	0.281	Experience
	17. The GP asks me if I am satisfied with the organisation of my care	0.497	0.408	Experience
	18. The GP contacts me to know how things are going	0.520	-0.012	Experience
**Factor 2**	1. The GP asks about my ideas when we made a treatment plan	0.468	0.573	Customisation
	3. The GP gives me a copy of my treatment plan	0.638	0.673	Cooperation
	4. The GP gives me choices about treatment to think about	0.596	0.684	Cooperation
	9. The GP asks me to talk about my goals in caring for my illness	0.551	0.654	Customisation
	11. The GP helps me to list things that I can do to improve my health	0.605	0.633	Cooperation
	12. The GP refers me to a dietician, health educator, or counsellor	0.384	0.719	Cooperation
	13. The GP encourages me to go to a specific group to help me with my chronic illnesses	0.252	0.888	Cooperation
	14. The GP encourages me to attend programs in the community that could help me	0.318	0.767	Cooperation

The resulting RMSEA value for this factorial solution was 0.083 with a 90% CI=[0.068;0.099], thus suggesting a ‘reasonable’ level of fit.

#### Reliability

Overall, the Cronbach alpha coefficient showed a somewhat high level of internal consistency for the 18-items French-PACIC questionnaire with a value close to 0.87. This measure was also used to test the internal consistency within each dimension and the values were respectively 0.81 and 0.84. The ordinal alpha coefficient led to the same conclusions about the reliability of the two dimensions with values respectively of 0.89 and 0.88.

### Implementation of the CCM in the French context

Overall the patients perceived the CCM as being moderately well implemented in their chronic care (Table 3), with a median PACIC score of 2. However this global assessment hides differences between the two dimensions of the questionnaire. The items pertaining to the 2^nd^ dimension "Collaborative chronic care" appeared to be less implemented than those of the 1^st^ dimension "Personalised chronic care", with median scores respectively of 3 and 1. However, the Wilcoxon test of signed rank suggested no statistically significant difference between the PACIC scores of these two dimensions (S = -342.5, P-value = 0.5096). These results were consistent with the French context in which the GP care is still mainly focused on acute conditions and therefore most of the CCM recommendations were not implemented. The French-version of the PACIC can be obtained from the authors on request.

The ANOVA showed a significant effect of age and the self-reported health (Table 6). The patients less than 65 years old had a mean composite score higher than those older than 65years, respectively 2.56 (SD=0.7) and 2.25 (SD=0.73). Interestingly, the patients who reported a somewhat good health state (i.e. good, very good, or excellent) showed PACIC scores larger than those who reported a deteriorated health state (i.e. satisfactory or bad), respectively 2.6 (SD=0.73) and 2.3 (SD=0.7).

**Table 6:** ANOVA of the PACIC score according to individual characteristics

**Characteristic**	**Degrees of freedom**	**Type III sum of squares**	**Mean square**	**P-value**
Number of chronic conditions (in class)	2	1.00	0.50	0.367
Number of GP consultations per year (in class)	2	0.42	0.21	0.657
Gender	1	0.09	0.09	0.676
Self-reported health	1	2.43	2.43	0.028
Age (in class)	3	1.70	1.70	0.019

Model: N = 147; degrees of freedom used = 9; R2 = 12.2%; P-value = 0.031.

## Discussion

This study has successfully translated and adapted the PACIC questionnaire to the French context. The psychometric properties of the French-version have been tested and results have provided support of its validity. The different analyses have left some issues that are discussed below.

### Why a structure of the questionnaire in 2 dimensions instead of 5?

The initial English-version of the PACIC consisted in 5 dimensions derived from the components of the chronic care model [17]. These dimensions were pre-specified rather than identified through data analysis. In line with this initial approach, most of the validation studies have used the confirmatory factor analysis (CFA) method to verify the adequacy of the data to this 5-dimensions structure. Among the 7 studies identified in the literature, 4 provided support for a 5-dimensions structure [24,27,37,38] and 3 suggested that such structure did not adequately fit the PACIC data [28,39,40]. As in our study, an alternative approach consisted in using an exploratory factor analysis (EFA) to emerge the number of dimensions from the data. In the literature this approach has been used in 5 studies, with 2 studies identifying 5 dimensions [25,41] and the 3 others studies identifying 1 or 2 dimensions [28,42]. Our 2-dimension structure of the PACIC is more closely related to these last validation studies which included items in more general dimensions. At the extreme situation, Gugiu et al. discussed the potential of PACIC data to emerge only one dimension [28]. These differences of structure between the validation studies may arise from methodological differences between the studies. The studies validating the PACIC survey are quite heterogeneous in the way they performed the factor analysis (e.g. confirmatory vs exploratory analysis; Pearson correlation vs Spearman or Polychorric correlation as input matrix; Orthogonal vs Oblique rotation of the factors; Kaiser criterion + Scree plot vs Parallel analysis to select the factors). As in Gugiu et al. [28], this study relied on non-standard, but theoretically more appropriate, methodological choices (i.e. Oblique rotation; WLS estimator; Parallel analysis; Exploratory/Confirmatory analysis) and raises doubt about the structure of the PACIC in 5 dimensions. These factorial differences might also arise from true differences between the health systems and the sample of patients in which the studies took place. Some studies are especially interested on the perspective of patients suffering from some chronic condition, whereas other studies focused on patients suffering from multiple chronic conditions, like our study.

### Why a version of the questionnaire in 18 items instead of 20?

Merging 4 items into 2 was a direct consequence of the active role played by respondents in the adaptation of the PACIC questionnaire. They merged items perceived as redundant. Second, the removal of two items with very high level of "*Never*" responses suggested that these items were not appropriate to describe the way chronic conditions were managed in primary care in France. As previously discussed by Rick et al. (2012), Wensing et al. (2008) and Spicer et al. (2010), the PACIC questionnaire has been firstly developed in a specific context, namely the United States, where the organization of the healthcare system and current initiatives in terms of chronic disease management may differ from other countries [18,39,41]. Then in terms of face validity it was deemed necessary to adapt the PACIC questionnaire to each context in which it was transposed to adequately reflect the experience of the patients. Other studies have also removed or reframed some initial PACIC items during the adaptation/translation process. McIntosh (2008) suggested a 17-items version [40] and Gugiu et al. have developed a valid short-form of the PACIC questionnaire in 11 items [19].

### What about the relationships of the PACIC scores with individual characteristics?

In our study, we found significant relationships between the overall PACIC score and two patients’ characteristics, namely the age and the self-reported health. These results are partly in line with the literature. In terms of self-reported health, 2 studies have previously investigated the relationship with the PACIC score(s) and concluded to significance [37,42]. In terms of age, the evidence is mixed: in the 9 studies which tested the relationship with PACIC score, only 3 showed a significant result [38,39]. Regarding the other characteristics tested in our study, the results are also in line with the literature: 5 studies out of 7 found no relationship between number of consultations and PACIC score, and 7 studies out of 10 found no relationship between the gender and PACIC score. Only one study has previously analyzed the effect of the number of chronic conditions and did not conclude there was a significant difference [37], like us. In our study, we have not investigated potential differences in PACIC scores according to the type of chronic conditions. However, Glasgow et al. have tested the initial English-version of the PACIC in populations with various chronic conditions and displayed no differences in its psychometric properties across these conditions [17]. Developers of the chronic care model argued that this model was generic and could be successfully applied to variety of chronic conditions [11,43].

### What about the implementation of the chronic care model?

According to the overall PACIC score, our results appear to be consistent with other studies performed in different countries and chronic conditions. We identified 17 studies that reported an overall PACIC score which ranged from 2.4 [39] to 3.2 [17] with a median of 2.84. With an overall PACIC score of 2.6, our study is in line with the studies of Vrjihoef et al. and Glasgow et al. respectively performed on 89 and 266 patients suffering from multiple chronic conditions in the Netherlands and in USA [24,44]. The comparison of our result with those of Szecsenyi et al. is especially interesting, because French and German healthcare systems shared common organizational features [45]. In addition Szecsenyi et al. used the PACIC questionnaire in two populations of diabetes patients, one who participated in a disease management programme and the other not. They showed a significant difference of overall PACIC score between these two groups of patients (i.e. 3.21 vs 2.86). A similar disease management programme is currently implemented in France and we assume that similar pattern of results should be identified using this French-version of the PACIC questionnaire.

### Limitations

It is important to note that our study has some limitations. First, the primary objective of this study was to validate the PACIC survey in the French language on a sample of patients suffering from multiple chronic conditions. For this purpose, we selected patients suffering from the OSAS as a well-designed population for this study because they are usually suffering from other chronic conditions (e.g. diabetes, hypertension, arthritis) and they experience complex medical care involving many health professionals in different places. Despite this, we used selective inclusion criteria to recruit only patients with a stabilized OSAS (i.e. asymptomatic condition and treatment well accepted by the patients), we cannot preclude any potential influence on our results. Instead of comparing results across different chronic conditions, for future research on the PACIC, it would be interesting to compare the ratings after controlling for patients’ ease of interaction with the healthcare system (i.e. number of consultation, self-perceived difficulty to get the needed service) and self-perceived health condition. Second, the sample size of recruited patients can be considered as being on the lower bound of acceptability. Despite being in line with a part of the international literature with a 5:1 subject to variable ratio, conventional recommendations are closer to 10:1. Moreover, these recommendations are based on continuous data, which we do not have. The ratio for ordinal data is higher. Thus our sample size might be too restrictive for further analyses, for example, based on structural equation modeling. However our results are still in accordance with part of the PACIC literature using same analytical approach, thus providing confidence in our results.

Third, retest reliability was not examined. To date only three studies have investigated this issue and showed mixed evidence according to intervals (i.e. 2 weeks, 3 or 8 months) used between the two measurement points [17,19,25]. On a sample of 56 patients, Rosemann et al. [25] showed a good reproducibility of PACIC scores using a 2-weeks interval (r = 0.81). On a sample of 250 patients and using a short-version of the PACIC survey, Gugiu et al. [19] showed a reasonable test-retest reliability at 8 months (r = 0.638).

Fourth, we have not tested the concurrent (or external) validity of our adaptation, because we lack of patient surveys developed and validated in French language. Wensing et al. explored this issue using the EUROPEP instrument and they showed strong correlations between PACIC and EUROPEP scales [41].

Fifth, we have not tested the sensitivity to changes of the PACIC ratings. Few studies have investigated this issue and showed mixed evidence. Maindal et al. investigated the sensitivity to change (i.e. enrollment in a disease management programme) of the PACIC among 585 patients with chronic vascular diseases and showed that the PACIC scores improved significantly [37].

## Conclusion

In this study, we have successfully translated, culturally adapted and validated a French-version of the PACIC questionnaire. This tool may now be used in population of patients with multiple chronic conditions to measure the level of CCM achievement from their perspective, and to monitor the quality improvements realized by the medical structures, mainly in general practice.

## Competing interests

The authors declare that they have no competing interests.

## Authors’ contribution

NK participated in the design of the study, in the statistical analysis of the data, and in the writing of the manuscript. MLV participated in the design of the study and in the statistical analysis of the data. NPF participated in the design of the study and in the writing of the manuscript. All authors read and approved the final manuscript.

## Pre-publication history

The pre-publication history for this paper can be accessed here:

http://www.biomedcentral.com/1472-6963/14/269/prepub
